# Cardiac Autonomic Neuropathy: A Progressive Consequence of Chronic Low-Grade Inflammation in Type 2 Diabetes and Related Metabolic Disorders

**DOI:** 10.3390/ijms21239005

**Published:** 2020-11-27

**Authors:** Nour-Mounira Z. Bakkar, Haneen S. Dwaib, Souha Fares, Ali H. Eid, Yusra Al-Dhaheri, Ahmed F. El-Yazbi

**Affiliations:** 1 Department of Pharmacology and Toxicology, Faculty of Medicine, American University of Beirut, Riad El-Solh 1107 2020, Beirut 11-0236, Lebanon; nb87@aub.edu.lb (N.-M.Z.B.); hsd12@mail.aub.edu (H.S.D.); ae81@aub.edu.lb (A.H.E.); 2 Rafic Hariri School of Nursing, American University of Beirut, Riad El-Solh 1107 2020, Beirut 11-0236, Lebanon; sf31@aub.edu.lb; 3 Department of Basic Medical Sciences, College of Medicine, QU Health, Qatar University, Doha 2713, Qatar; 4 Biomedical and Pharmaceutical Research Unit, QU Health, Qatar University, Doha 2713, Qatar; 5 Department of Biology, College of Science, United Arab Emirates University, Al-Ain 15551, UAE; 6 Department of Pharmacology and Toxicology, Faculty of Pharmacy, Alexandria University, Alexandria 21521, Egypt

**Keywords:** cardiac autonomic neuropathy, inflammation, reactive oxygen species, type 2 diabetes

## Abstract

Cardiac autonomic neuropathy (CAN) is one of the earliest complications of type 2 diabetes (T2D), presenting a silent cause of cardiovascular morbidity and mortality. Recent research relates the pathogenesis of cardiovascular disease in T2D to an ensuing chronic, low-grade proinflammatory and pro-oxidative environment, being the hallmark of the metabolic syndrome. Metabolic inflammation emerges as adipose tissue inflammatory changes extending systemically, on the advent of hyperglycemia, to reach central regions of the brain. In light of changes in glucose and insulin homeostasis, dysbiosis or alteration of the gut microbiome (GM) emerges, further contributing to inflammatory processes through increased gut and blood–brain barrier permeability. Interestingly, studies reveal that the determinants of oxidative stress and inflammation progression exist at the crossroad of CAN manifestations, dictating their evolution along the natural course of T2D development. Indeed, sympathetic and parasympathetic deterioration was shown to correlate with markers of adipose, vascular, and systemic inflammation. Additionally, evidence points out that dysbiosis could promote a sympatho-excitatory state through differentially affecting the secretion of hormones and neuromodulators, such as norepinephrine, serotonin, and γ-aminobutyric acid, and acting along the renin–angiotensin–aldosterone axis. Emerging neuronal inflammation and concomitant autophagic defects in brainstem nuclei were described as possible underlying mechanisms of CAN in experimental models of metabolic syndrome and T2D. Drugs with anti-inflammatory characteristics provide potential avenues for targeting pathways involved in CAN initiation and progression. The aim of this review is to delineate the etiology of CAN in the context of a metabolic disorder characterized by elevated oxidative and inflammatory load.

## 1. Introduction

Cardiac autonomic neuropathy (CAN) is one of the earliest manifestations of type 2 diabetes (T2D). It constitutes the major cause of silent cardiovascular events in patients without overt cardiac disease. The high prevalence of CAN in patients newly diagnosed with T2D [1] suggests that its pathophysiology is rooted in an earlier stage of metabolic derangement, possibly being prediabetes. Recent knowledge about the nature of disease progression has led researchers to study the status of CAN in patients with recent-onset diabetes. Interestingly, comparisons between type 1 and type 2 diabetic individuals further confirm the fact that CAN processes in T2D start earlier than the onset of overt metabolic impairment [2]. To this end, guidelines recommend CAN screening in T2D patients as early as their first diagnosis as opposed to 5 years after onset in T1D [3]. Thus, it follows that dysglycemia is not the exclusive cause responsible for the initiation of CAN and its progression in T2D. This is clearly reflected when comparing the risk factors of CAN in both diseases. Above poor glycemic control in T1D, obesity and its associated dyslipidemia, hyperinsulinemia, and hypertension (HTN) present additional risk factors for CAN in T2D [4]. Hence, different factors in the etiology of the disease are shown to contribute differentially to CAN manifestations. 

Two types of autonomic dysfunction can be associated with diabetes, either intrinsic or extrinsic [5]. The first is related to an insult caused directly to autonomic nerves, whereas the other can be secondary to cardiovascular dysfunction, such as dilated cardiomyopathy and aortic stiffness. Studies concerned with investigating the major contributors to cardiac autonomic dysfunction in T2D have indicated that it is primarily intrinsic in nature [6,7].

Clinically, different tests are used to assess CAN and the function of the different arms of autonomic control. These include, but are not limited to, the Valsalva maneuver (Table 1), deep breathing, and lying to standing—which constitute the Ewing battery tests—and orthostatic hypotension. While the latter was shown to be indicative of the integrity of sympathetic function, the former are reflective of the cardiovagal status. Various parameters can be calculated from CAN assessment tests. These represent the indices of heart rate variability (HRV) (Table 2) and baroreceptor/reflex sensitivity (BRS), and include various time- and frequency-domain parameters [5]. While time-domain parameters reflect the degree of dispersion in a dynamic timeseries (of heart rate (HR) or blood pressure (BP)), frequency-domain parameters separate a particular waveform into its three major components: high, low, and very low frequency (HF, LF, and VLF, respectively) [5]. While HF was shown to be indicative of parasympathetic function and VLF of sympathetic integrity, LF has been tied to both sympathetic and parasympathetic functionality.

Technically, parameters of BRS quantify the change of HRV with respect to BP variability. BRS reflects the ability of baroreceptors in carotid sinus and aortic arch to detect changes in aortic distention and relay them to brainstem nuclei responsible for cardiac autonomic control [9]. Specifically, the nucleus of tractus solitary is responsible for parasympathetic discharge, while the rostral ventrolateral medulla drives sympathetic outflow [10]. In fact, two checkpoints, mediated by the status of baroreceptors, can determine the strength of the relationship between changes in BP and reflex modulation of HR. The first corresponds to physical properties of the vascular system carrying pressure signals to baroreceptors, while the second represents the major effector in the autonomic nervous system. The latter is referred to as neuronal BRS while the former is referred to as mechanical BRS. Studies in T2D patients have indicated that autonomic neuropathy is a stronger determinant of BRS than carotid stiffness [6]. To this end, different studies have turned their attention toward the characterization of changes in the different ganglia and nuclei in the cardiac neural network in metabolic syndrome [11], prediabetes [12], and T2D [13,14,15].

## 2. Rationale 

This review focuses mainly on the determinants of CAN on the progression from prediabetes to T2D. It also describes a temporal framework for the different manifestations of such a complication, explaining how multiple disease components can have differential impacts on symptoms. While innumerable studies have investigated the effect of these different factors in various disease conditions, a significant need remains to examine them collectively along the natural history of T2D. Our aim was to review these changes in T2D subjects and animal models with a focus on the role of local and systemic inflammatory changes and their ensuing impact on peripheral and central effectors including neuronal oxidative stress and autophagy.

## 3. The Metabolic Syndrome: A Continuum of Low-Grade Pro-Oxidative and Proinflammatory Processes 

Current understanding of the metabolic syndrome reveals the presence of an inflammatory component. Different mechanisms in the course of progression to T2D trigger the initiation of inflammatory processes that are varied in nature but are essentially linked [16]. The so-called “metabolic inflammation” (also known as meta-inflammation) distinguishes T2D from T1D. Interestingly, a population-based study comparing inflammatory profiles in normoglycemic, prediabetic, and T2D individuals offered a spectrum of differential change in inflammatory biomarkers with disease progression [17]. 

### 3.1. The Role of Altered Glucose Homeostasis in Meta-Inflammation

In the prediabetic stage, changes in glucose and insulin homeostasis have been shown to be linked to inflammation pathogenesis even before the advent of hyperglycemia [18,19]. An increase in insulin demand and production secondary to insulin resistance is accompanied by elevated pancreatic endoplasmic reticulum stress initiating pro-oxidative and proinflammatory processes [20,21]. Additionally, hyperinsulinemia-induced lipid storage was shown to promote adipose tissue-specific inflammation and a subsequent acute phase response [22,23]. This was shown to be mediated by adipose tissue expansion promoting hypoxia of poorly vascularized tissues, which constitutes the driving force for the activation of nuclear factor-κB (NF-κB), a sensor of oxidative stress [24]. On the activation of NF-κB, adipose tissues secrete proinflammatory cytokines such as interleukin-6 (IL-6) and tumor necrosis factor α (TNF-α), which promote liver synthesis of acute phase proteins such as C reactive protein (CRP) and plasminogen activator inhibitor-1 (PAI-1) [25,26]. Consequently, adipose tissue hypertrophy leads to apoptosis attracting macrophages in crown-like structures [27,28]. Immune cells release reactive oxygen species (ROS) in response to cytokine upregulation [29]. Moreover, overnutrition overwhelms inherent mitochondrial capacity for scavenging excess ROS produced by metabolic processes promoting further upregulation of proinflammatory processes through NF-κB pathways [30]. On the onset of hyperglycemia, however, elevated mitochondrial aerobic respiration and activity of the electron transport chain, as well as advanced glycated end products, aggravates oxidative stress, which presents another activator of inflammatory cascades mediated by NF-κB, cAMP-regulated element-binding protein, and activator protein 1 [31]. Additionally, neurohormonal stimulation by the renin–angiotensin–aldosterone system (RAAS) was shown to play a role in aggravating oxidative stress and inflammation [32]. 

Interestingly, metabolic inflammatory processes are evident in the cardiovascular, neuronal, and neurovascular systems, indicating their possible involvement in the etiology of cardiac autonomic dysfunction in the metabolic syndrome [33,34,35]. Hypoxia driven by vascular dysfunction activates immune cells of the central nervous system, producing cytokines such as IL-1β, which in turn triggers effectors downstream of NF-κB further producing cytokines such as IL-6. Additionally, the metabolic syndrome is a known disrupter of the integrity of the blood–brain barrier (BBB) via altering the permeability of the choroid plexus [36,37]. This was attributed to increased ROS production leading to decreased expression of tight junction proteins. Hence, it promotes infiltration of proinflammatory cytokines and immune cells from the bloodstream to the central nervous system, especially in the context of systemic inflammation characteristic of T2D. The latter contributes to compromising BBB functions by increasing the permeability of the basement membrane of the BBB, via matrix metalloproteases [38], allowing for immune cell extravasation and upregulating leukocyte adhesion molecules, such as intracellular adhesion molecule-1 (ICAM-1), vascular cell adhesion molecule-1 (VCAM-1), P-selectin, and E-selectin [37]. In fact, increased oxidative stress in the diabetic brain is related to decreased antioxidant defense enzymes and molecules concomitant with an increase in the polyol pathway resulting in a decrease in NADPH recycling [39]. It was also shown that hyperinsulinemia can lead to increased neuronal oxidative stress through decreased mitochondrial PI3K/Akt signaling pathway [40]. Such changes were shown to be associated with autophagic disturbances in different peripheral and central neurons [39,41,42]. Alternatively, accumulation of ROS-generating mitochondria resulting from autophagy suppression could activate, in addition to NF-κB, the NLRP3 inflammasome responsible for proinflammatory cytokine maturation [39]. Additionally, mitochondrial oxidative damage was shown to be accompanied by a decrease in ATP levels resulting from suppressed mitochondrial energization potential. Hence, the aforementioned changes could ultimately lead to neuroinflammation.

### 3.2. Contribution of Gut Microbiota to Meta-Inflammation

Alterations in the gut microbiome (GM) or dysbiosis has been recently linked to many morbidities, such as metabolic and immune-related disorders [43]. The GM community can affect the host health via two routes: the bacterial components or pathogen-associated molecular patterns (PAMPs), including cell-wall constituents such as lipopolysaccharides (LPS) [44], and the metabolites produced when digesting and processing food in the gut. Hence, dysbiosis outcomes depend on the bacterial Phyla alterations in the gut [45]. Moreover, GM plays a vital role in regulating the permeability of intestinal mucosa [46]. GM manipulates the host’s metabolism; hence, dysbiosis was found to be linked to some compromised metabolic states and related diseases [47].

One of the major contributors to dysbiosis is dietary intake. A high-fat diet (HFD), implicated in the production of the metabolic challenge leading to metabolic syndrome and T2D, promotes an increase in serum LPS. This was proposed to occur due to increased permeability of the gut by the microbiota, which is linked to metabolic endotoxemia. LPS acts through the Toll-like receptor 4 (TLR4) signaling pathway, where TLR4 is expressed on macrophages and adipose tissue and is activated upon LPS recognition. The LPS/TLR4 complex has two main signaling pathways: the MyD88-independent pathway that gives rise to Type 1 interferons (IFNs) and the MyD88-dependent pathway that activates proinflammatory cytokines such as IL-1, IL-6 and TNF-α. Both pathways act via NF-κB [48,49,50]. Thus, upon activation, this complex stimulates white adipose tissue inflammation and proinflammatory macrophage infiltration and is also linked to an increase in monocyte chemoattractant protein-1 (MCP-1) [51]. 

In addition to that, work on mice tissues showed that an HFD enhanced the transport of LPS through chylomicrons, rather than the traditional paracellular pathway [52,53]. Increased gut permeability, endotoxemia, and fatty liver were also observed in animal models fed a high-fructose diet, HFD, or both [44]. Upon the discovery of the importance of the gut microbiota in dictating metabolic status, different nutritional supplementations were introduced in an attempt to manage the colonic composition of bacteria. These are pre-, pro-, and postbiotics [54]. Whereas probiotics are essentially exogenous microbial supplements aimed at optimizing microbial balance, their effects are rather transient. Prebiotics, on the other hand, are nondigestible food ingredients, such as fructo-oligosaccharides, which promote the growth/activity of select endogenous bacteria residing in the gut [55]. Alternatively, postbiotics are the metabolites produced by probiotics, specifically lactic acid bacteria [54]. Interestingly, animals treated with pre-, pro- and postbiotics showed an improved integrity of the intestinal barrier [56,57,58] and, consequently, a reduction in endotoxemia, fatty liver, glucose intolerance, and obesity [59]. Hereafter, dysbiosis often leads to increased gut permeability through reduced expression of tight junction proteins and mucins [60] or the consumption of gut mucus by the GM in the absence of dietary fiber intake [59]. 

Moreover, the type of fat in the diet determines the changes in GM community. For example, some research showed that unsaturated fatty acids such as fish oil increased *Lactobacillus* abundance in the gut, which is known to be correlated with lean phenotype and reduced adipose tissue inflammation and macrophage infiltration. *Akkermansia muciniphila* and *Bifidobacterium* showed similar positive effects to *Lactobacillus*. On the other hand, a saturated-fat diet is highly linked to an increase in some taxa such as *Bilophila*, *Bacteroides*, and *Turicibacter,* which in turn appear to increase adiposity, adipose tissue inflammation, insulin insensitivity, and TLR4 activation. Furthermore, these taxa were found to activate the MyD88-dependent pathway among lard-fed animals and were correlated with increased inflammation of white adipose tissue and size of adipocytes [45,49,61]. This was confirmed in MyD88-deficient mice fed a lard-rich diet, as these mice had smaller adipocytes and did not develop obesity, compared to their wildtype lard-fed littermates [49]. Moreover, a deletion of MyD88 was associated with reduced macrophage inflammatory response and aortic lesions [45].

A Gram-negative microbiota was proposed to exacerbate the high-fat effect in murine models via the TLR4 pathway. Interestingly, TLR4 knockout (KO) models fed an HFD had intact gut integrity compared to wildtype HFD-fed mice [62]. Moreover, TLR4 KO mice showed a resistant phenotype to cardiovascular diseases [63]. According to some evidence, dysbiosis is correlated to paracellular permeability, which is associated with compromised expression and localization of tight junction proteins in the epithelial layer, especially occludin and zonula occludens-1 in the small intestine [43]. 

Two worthily mentioned metabolites that have major contribution to cardiovascular insults are short-chain fatty acids (SCFAs) and trimethylamine oxide (TMAO). In general, these two metabolites have opposing effects in terms of metabolic and cardiovascular state [63,64]. SCFAs, mainly butyrate, acetate, and propionate, are produced by plant polysaccharide-fermenting bacteria, such as *Lactobacillus* and *Bifidobacterium* [63,64], which are compromised with the increase in Firmicutes-to-Bacteriodetes ratio [65]. The decrease in SCFAs and their producing bacteria was found to be correlated to HTN and other cardiovascular insults [63,65,66]. Furthermore, SCFAs seem to be associated with enhanced gut barrier function, by increasing the expression of mucin and tight junctions proteins [60,67]. An increase in TMAO is persistently associated with an increased risk of wide array of cardiovascular diseases in T2D and nondiabetic subjects [68], such as atherosclerosis and ischemic heart failure [63]. TMAO is synthesized in the liver from trimethylamine (TMA), a product of choline digestion by gut bacteria, via flavin-containing monooxygenase 3 (FMO3) [64,69,70]. Of note, elevated levels of TMAO are associated with T2D by affecting insulin sensitivity and disrupting its signaling pathway through HFD-induced adipose tissue inflammation [69]. Furthermore, TMAO is linked to endothelial dysfunction by increasing inflammation and adhesion factors [66] and altering endothelial nitric oxide synthase (eNOS) function and nitric oxide (NO) production [64,65]. Moreover, TMAO was found to be involved in activating inflammasome complex in vascular cells and stimulating ROS production [71], in addition to its proatherogenic role [45,71].

Interestingly, GM has been implicated in the control of adipose tissue expansion and inflammation [72] and can consequently be a determinant of systemic inflammation in metabolic diseases such as T2D. Along the same lines, dysbiosis could disrupt the BBB by downregulating endothelial tight junctions, thus promoting neuroinflammation [37].

## 4. Progression of CAN: From Metabolic Syndrome and Prediabetes to T2D

### 4.1. Determinants of CAN in Early-Onset and Advanced T2D

Comparisons with status and manifestations of CAN in T1D implicate different disease-specific characteristics in the initiation and progression of CAN in T2D. Studies conducted by Ziegler et al. assessed the status of CAN in patients with recent onset diabetes, i.e., less than or equal to 1 year after their first diagnosis [2,73]. Their results provide indication that the pathophysiologic trigger of CAN in recent-onset T2D is independent of hyperglycemia but rather tied to metabolic characteristics related to obesity (body mass index (BMI) > 30, central obesity, and increased fat mass) and dyslipidemia, distinguishing this population from their control, and subsequently T1D, counterparts [2]. Indeed, T2D but not T1D patients presented with early parasympathetic and sympathetic blunting compared to controls, independently correlated with insulin resistance (M-value of whole-body insulin sensitivity and T2D; see Table 3). It is worth mentioning, however, that, although this population of T2D patients had elevated systolic BP and HR compared to control (healthy, age-matched subjects), they did not show signs of sympathetic augmentation on HRV assessment, i.e., LF and LF/HF of HRV were not different from controls [2]. Interestingly, these patients showed only a slight increase in HbA1c, potentially ruling out a contribution of glycated end products in the observed phenotype. In particular, unlike T1D patients, recent-onset T2D patients showed hyperinsulinemia, and increased high-sensitivity CRP [2], indicating a possible mechanism for hyperinsulinemia-induced systemic inflammation in the pathogenesis of premature baroreceptor dysfunction. Yet, no correlations among hyperinsulinemia, systemic inflammation, and early baroreceptor blunting, especially sympathetic, were studied in this T2D patient population. 

Interestingly, different studies have brought into the picture another factor in the pathogenesis of CAN in early-onset T2D; this is oxidative stress related to acute glycemic excursions, rather than chronic hyperglycemia. On progress to T2D, changes in glucose tolerance and insulin sensitivity take the form of glycemic variability. Importantly, glycemic variability was shown to have the power of predicting CAN in recent onset, where average glucose level failed [75]. Specifically, glycemic variability was higher in T2D patients with CAN, according to Ewing battery tests, than in those without CAN. A role for systemic oxidative stress in the initiation of early parasympathetic dysfunction was, thus, proposed. This particularly pertains to endothelial dysfunction [76,77] and eventually baroreceptor impairment. In fact, increased ROS production in early metabolic insults was shown to be related to decreased endothelial-dependent hyperpolarization secondary to reduced expression of potassium inward rectifier channels [78]. Such an increase is presumed to bring about elevated vascular tone through impairing eNOS activity, ultimately diminishing NO-induced vasorelaxation [79]. Moreover, a study assessing the relationship between endothelial dysfunction and CAN revealed a positive association between NO and eNOS and measures of cardiovagal control, presenting determinants of endothelial function as biomarkers for the pathogenesis of parasympathetic neuropathy in T2D patients [77].

Interestingly, a study assessed the effect of glycemic variability on BRS in T2D patients and again revealed that its elevation is independently correlated with decreased BRS [80]. Above the detrimental effects of oxidative stress on endothelial function and neuropathy, the study presumed that hyperinsulinemia caused by acute fluctuations in blood glucose could be responsible for the observed blunted BRS [80]. Additionally, results showed that BRS decreased with diabetes duration, indicating worsening status with progression of disease components. However, the study did not distinguish between the different arms of the baroreflex control and, thus, could not specify whether sympathetic or parasympathetic deterioration was responsible for this drop in sensitivity. Indeed, a study assessing the effect of glycemic control and disease duration on HRV in T2D patients revealed that worse glycemic indices and longer duration were accompanied by lower parameters of both sympathetic and parasympathetic determinants of HRV [81]. The impact of glycemic control on CAN could be partially explained by a reduction in antioxidant effectors and increase in prooxidative pathways, leading to neuronal ischemia and subsequent damage [82]. However, the contribution of hyperglycemia to inflammatory biomarkers in T2D cannot be overlooked [83]. Thus, it could be through an exaggerated inflammatory state that hyperglycemia worsens the status of CAN with disease progression [84].

### 4.2. Effect of Glucose Homeostasis along the Continuum of Prediabetes to Early-Onset T2D on CAN

In the same way, comparisons between prediabetic and type 2 diabetic manifestations of CAN allow us to draw conclusions about the pathophysiology of CAN development over the natural course of the disease. Hyperinsulinemia secondary to peripheral insulin resistance is the hallmark of the prediabetic stage [85]. However, the superimposition of hyperinsulinemia with sympathetic augmentation makes it unclear which causes the other. A study of a fructose-induced glucose intolerance in mice revealed that sympathetic augmentation (elevated LF of BP variability) and autonomic imbalance (increase in LF/HF) appear before hyperinsulinemia and other metabolic derangements in the course of the metabolic syndrome [86]. Major research has been channeled to the study of early sympathetic augmentation, vagal withdrawal, and altered sympatho-vagal balance; however, little remains known about the origin and etiology of sympathetic dysfunction at a later stage of the disease. While chronic hyperglycemia appears to be the eliciting factor, orthostatic hypotension was thought to be brought about by damage of efferent vasomotor neurons in splanchnic blood vessels [87]. Moreover, earlier studies reported on cardiac sympathetic denervation [88,89]. Little remains discovered, however, about the status of central control of sympathetic activity or central sympathetic neuropathy.

Prior studies demonstrating the presence of early processes culminating in CAN in the metabolic syndrome examined the correlation of different stages of impaired glucose homeostasis, defined by isolated impaired fasting glucose (iIFG) and impaired oral glucose tolerance (IGT), as opposed to subjects with normal glucose tolerance (NGT) and newly diagnosed diabetics (NDD) (Table 4), with cardiac autonomic control [8]. In one study, CAN was assessed using two BRS parameters. The first was the Valsalva ratio, corresponding to the longest R–R interval resulting from parasympathetic activation over the shortest R–R interval resulting from sympathetic activation, while the second was α_LF_, an index of BRS in the LF region corresponding to sympathetic activity [90]. Results showed that, in the absence of obesity, there is an early decrease in α_LF_ of BRS in patients with IGT only, which is further complicated by a blunting of the Valsalva ratio in borderline overweight NDD, confirming a deterioration of sympathetic function. Different speculations about the origin of the difference in BRS with nondiabetics were made, including differences in the site (i.e., hepatic vs. muscular) and severity of insulin resistance. However, the levels of blood insulin and inflammatory markers in these different conditions were not given. Importantly, a study by Festa et al. reported a consistent independent correlation between insulin resistance, as revealed by frequently sampled glucose tolerance test, and inflammation among patients with insulin resistance syndrome (IRS), NGT, and IGT [18]. Fasting insulin and proinsulin were also associated with chronic subclinical inflammation in these three categories [18], and elevated CRP levels in T2D patients were shown to be correlated with reduced cardiovagal function [77]. It can, thus, be presumed that insulin-resistance-associated hyperinsulinemia could be at the origin of inflammation producing CAN in patients with IGT.

### 4.3. Influence of Obesity Indices and Dyslipidemia

When studying CAN in prediabetic and T2D patients, it is important to acknowledge the status of obesity in assessed individuals. Indeed, studies investigating the relationship between obesity and CAN revealed that various factors differentially contribute to the pathogenesis of CAN. In nondiabetic obese men, percentage body fat, waist circumference, and visceral adipose tissue volume were associated with measures of reduced HRV, with percentage body fat correlating with the greatest number of HRV parameters [92]. In fact, obesity was shown to predict development of systemic inflammation [93]. Interestingly, hyperleptinemia was shown to mediate the relationship between visceral fat accumulation and CAN in T2D patients [94]. Additionally, dyslipidemia in the presence of obesity aggravates the blunted baroreflex control in T2D and makes it more resistant to lipid-lowering treatment otherwise effective in nonobese T2D patients [95]. It is noteworthy that dyslipidemia could have detrimental effects on CAN by exacerbating systemic inflammation [83].

## 5. Association between Adipose, Vascular, Systemic, and Neuroinflammation and CAN

**Figure 1:**
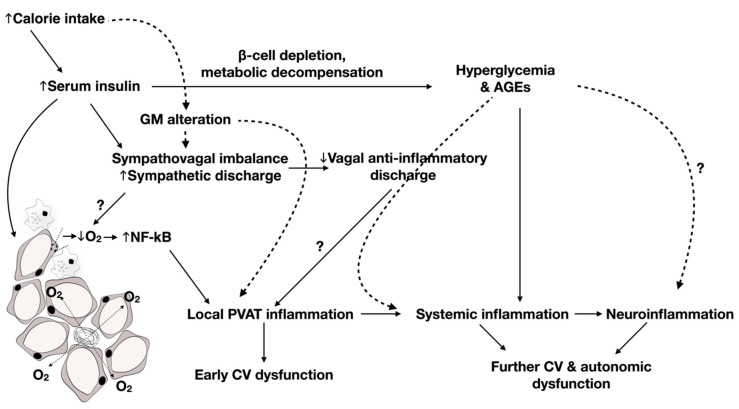
A possible mechanistic and temporal framework for cardiac autonomic neuropathy (CAN) progression in deteriorating metabolic function and the different interdependent pathways contributing to it. Increased caloric intake induces insulin resistance and alterations in gut microbiota (GM). The resulting hyperinsulinemia and dysbiosis could precipitate adipose inflammation directly or indirectly through sympatho-vagal imbalance. In this context, nuclear factor kappa B (NF-κB)-induced inflammation results from hypoxia related to perivascular adipose tissue (PVAT) expansion, as well as activation of Toll-like receptors 4 (TLR4) in response to metabolic endotoxemia. Increased proinflammatory sympathetic outflow and diminished anti-inflammatory vagal discharge could further contribute to PVAT inflammation. Later, metabolic decompensation results in β-cell depletion, hyperglycemia, and advanced glycated end products (AGEs), which in turn aggravate the pre-existing localized inflammation, triggering wider changes. Several markers of systemic inflammation have been shown to be associated with deterioration of cardiac autonomic control. Disruption of blood–brain barrier function associated with dysbiosis and systemic inflammation promotes spillage of proinflammatory cytokines to regions of the central nervous system resulting in neuroinflammation and further cardiac autonomic and cardiovascular (CV) dysfunction.

As an earlier study implicated hyperinsulinemia rather than insulin resistance in the pathogenesis of CAN, particularly impaired BRS [74], and hyperinsulinemia was shown to be the instigating cause of adipose inflammation independent of obesity [22], it can be speculated that it is through adipose inflammation that hyperinsulinemia affects cardiac autonomic control in the metabolic syndrome. Yet, autonomic, particularly sympathetic, function tends to deteriorate as diabetes progresses. Indeed, a study by Lieb et al. (2012) revealed a particularly decreased total spectral power (TSP), indicative of overall control of HRV in patients with established T2D, which was not otherwise present in newly diagnosed diabetics, who showed isolated parasympathetic blunting [96]. In fact, the strongest positive correlation was found to be present between total adiponectin-to-leptin ratio and TSP, indicating a contribution for these counteractive adipokines in dictating sympathetic tone. Additionally, increased PAI-1 was shown to be essentially increased in patients with established T2D compared to those with newly diagnosed diabetes (within 6 months of diagnosis). As such, one can conclude that, while prolonged exposure to hyperglycemia might underlie the observed CAN deterioration in T2D, this seems to occur through exacerbation of adipose tissue inflammation occurring in earlier stages of the disease. Later, Herder et al. (2017) retested the association between inflammation and CAN, especially in patients with new-onset T2D [97]. In this study, they found that the association between IL-6 and cardiac autonomic reflex tests was rather explained by confounding factors. This is in line with the results of a longitudinal study indicating that the association between baseline IL-6 levels and follow-up HRV measures was dependent on BMI [98], again potentially implicating adipose tissue expansion and inflammation. However, independent inverse associations were found between soluble adhesion molecules such as soluble ICAM and E-selectin and sympathetic and parasympathetic function, respectively, indicating a role for vascular inflammation in CAN [97]. Significantly, our previous studies examined the evolution of inflammatory changes in association with worsening of CAN as the metabolic insult progressed. We show that early prediabetic parasympathetic dysfunction is associated with perivascular adipose tissue inflammation [99]. After the development of hyperglycemia, localized adipose tissue inflammation degenerated into systemic inflammation as evident by increased serum IL-1β and signs of disseminated cardiovascular damage that were associated with increased neuronal oxidative stress, inflammation, and suppressed autophagy in the brainstem with concomitant deterioration of CAN, including both sympathetic and parasympathetic functions [84]. The scheme below, thus, provides evidence of a continuum of events, starting with perivascular adipose inflammation as a priming mechanism for vascular inflammation leading to systemic inflammation, presenting a temporal framework for the pathogenesis and progression of CAN in T2D [84,99,100] (Figure 1).

The observed role for neuroinflammation was emphasized in the previous study by Herder et al. (2017) [97]. Two interesting findings were derived from this study. The first is that, in recent-onset T2D, there was a negative correlation among markers of neuroinflammation, IL-18, and vagal efferent function, indicating that neuroinflammation could be the basis of worsening parasympathetic function at this stage of the disease. However, it is worth noting that, although elevated soluble ICAM was shown to be linked to depressed sympathetic function, increased IL-18 was indicated in increased sympatho-vagal balance. This shows that, although sympathetic function might be blunted, it can continue to be predominant over parasympathetic activity. Accordingly, comparisons made between patients with T1D and T2D showed decreased sympathetic activity in T2D, as indicated by the lower LF power of HR. The other important finding is that, while significant associations were shown to exist between cardiac autonomic function and markers of inflammation, such correlations were inexistent in T1D. This was consistent with results in experimental animals where autonomic dysfunction and neuroinflammation were attenuated when T1D was induced by streptozotocin, as opposed to decompensated T2D developing on a background of adipose tissue inflammation [84].

Examination of neuronal function in light of such an inflammatory milieu revealed several pathways implicated in the pathogenesis of CAN in the progress from metabolic syndrome to T2D. With a compromised BBB function, neuroinflammation becomes evident as systemic inflammatory cytokines are transported across the BBB [101,102]. Interestingly, a study showed that delivery of IL-6 to the nucleus of tractus solitary of rats was associated with blunted reflex bradycardia to increased BP [103]. In our work, a change in the status of autophagy, where systemically induced neuroinflammation presented with markers of suppressed autophagy in the brainstem of type-2 diabetic rats, was associated with worsening parasympathetic BRS and emerging blunted sympathetic BRS [84]. Indeed, cell culture studies confirmed the assumption that a serum factor, rather than hyperglycemia, is involved in neuronal damage culminating in CAN in T2D [84,104,105]. Significantly, incubation of the differentiated neuroblastoma cell line with serum from T2D patients with neuropathy was capable of inducing apoptosis in a Ca^2+^-dependent K^+^-flux manner [104]. Further study indicated mitochondrial dysfunction and autophagy as a mechanism for serum-mediated neuropathy in T2D [105]. Our work suggests that systemic proinflammatory cytokines are responsible for emerging blunted tachycardic responses in T2D [84]. Indeed, treatment of sympathetic-like neurons with sera from T2D rats recapitulated changes seen in brainstem of these rats. Differentiated pheochromocytoma (PC12) cells presented with increased inflammation concomitant with autophagy suppression. These manifestations were not otherwise seen when treating PC12 cells with sera from prediabetic rats (fed a mild hypercaloric diet) or when challenging them with media containing high macronutrient content (high glucose, high free fatty acids and insulin, or high glucose and free fatty acids). 

It remains questionable, however, in the light of primary autonomic dysfunction characteristic of T2D, whether adipose inflammation results in CAN or is a consequence of it. A longitudinal study on independent population cohorts established consistent associations between autonomic activity and inflammatory maskers (CRP and IL-6). Specifically, the study revealed that baseline vagal activity predicted the levels of inflammatory markers on follow-up. It is worth mentioning, however, that this study was not done exclusively on diabetic patients [106]. In fact, a longitudinal study in T2D patients revealed an independent negative association between baseline IL-1 receptor antagonist and HR, indicating a possible role for IL-1 in the pathogenesis of CAN [98]. Additionally, it is important to present sympathetic dysfunction as a possible contributing mechanism to T2D inflammatory processes especially with the presence of evidence regarding the involvement of the sympathetic nervous system in the regulation of immune responses [107,108]. 

## 6. Relationship between Gut Microbiota and Autonomic Dysfunction in Metabolic Diseases

Indeed, autonomic control is affected by the GM population in a bidirectional association [109]. The vagus nerve is known to mediate the relationship between the gut and the brain [109]. As vagal nerve afferent fibers are capable of sensing GM metabolites and transducing sensory messages to the central nervous system, the vagus can additionally affect the profile of the GM through its cholinergic anti-inflammatory efferents. As such, conditions associated with altered or reduced vagal tone have been shown to be characterized by dysbiosis [109]. 

Moreover, an increase in the sympathetic outflow has been reported to be associated with reduced gut integrity and increased permeability by compromising tight junction proteins, promoting a proinflammatory state alongside dysbiosis [66]. In the spontaneously hypertensive rat model, it was reported that these changes initiate an elevation in BP [110]. This was supported by using acetylcholine esterase (ACE) inhibitors in those rats, which tempered sympathetic activation, lowered BP and improved gut integrity, and promoted eubiosis [110]. On the other hand, the state of dysbiosis and its proinflammatory byproducts centrally activate the sympathetic nervous system, which induces HTN and a proinflammatory state [65]. Another pathway is through the lack of sympathetic nervous system suppression metabolites. Interestingly, some bifidobacteria, found to be reduced in hypertensive patients, produce γ-aminobutyric acid, which has a sympatholytic effect [65]. Furthermore, some bacteria can produce serotonin, norepinephrine, which are prohypertensive and proatherogenic [65]. Indeed, some lactobacilli that are altered in dysbiosis can activate the vagal tone and its subsequent anti-inflammatory outcomes [65]. 

Hence, dysbiosis was found to promote autonomic imbalance and induce a sympatho-excitatory state, which has been long recognized to be proinflammatory [65,111]. Noteworthy, dysbiosis can activate RAAS [112,113], which in turn can promote vascular dysfunction via the angiotensin II (Ang II) pathway [66,113,114]. In this regard, Ang II-mediated HTN was not attained in a germ-free mouse model [115]. Furthermore, SCFAs were found to be protective against Ang II-induced BP and RAAS activation [115]. Alternatively, the angiotensin-converting enzyme 2/angiotensin 1–7 axis has been suggested to attenuate immune responses via modulation of GM composition [116]. Hence, it can be proposed that changes in the RAAS system such as those seen in the metabolic syndrome and T2D can predict inflammatory responses through the GM. Another phenomenon through which dysbiosis could alter cardiac autonomic control in T2D patients and promote sympathetic predominance is a fatty liver. Indeed, elevated liver fat has been associated with reduced cardiovagal tone and BRS in T2D [117]. While evidence exists to link changes in GM in metabolic disorders to adipose tissue and systemic inflammation and to possible alteration of cardiac autonomic function under the same circumstances, it is not clear how these factors interplay in the temporal framework for the development and progression of T2D.

## 7. CAN as a Possible Mediator of the Impact of Meta-inflammation on Cardiovascular Disease in the Continuum from Prediabetes to T2D

Current research provides links among the change in inflammatory profile along the continuum of the disease, the severity of CAN, and the risk of cardiovascular disease [17,118]. In particular, studies show than the progression of CAN manifestations exist at the crossroad between inflammatory processes and cardiovascular complications. A study by Grossmann et al. identified different patterns of change in biomarkers of immune and inflammatory responses along the continuum of the diabetes [17]. In particular, they differentiated between biomarkers, the levels of which gradually increase with worsening of metabolic control from normoglycemia, prediabetes, to T2D (e.g., IL-1R antagonist, IL-18, and monocytes), and others exclusively increasing in the progression to subclinical disease i.e., prediabetes (such as CRP). Importantly, such changes coincide with the natural history of CAN in T2D, whereby an initial increase in CRP marks an early reduction in cardiovagal tone characteristic of the prediabetic stage [77]. Later, a delayed systemic decrease in IL-1R antagonist along with a concomitant increase in neuroinflammatory IL-18 comes in parallel with further blunting of parasympathetic function on the onset of T2D, as previously described [97,98]. Interestingly, there was a gradual increase in cardiovascular risk factors and comorbidities from normoglycemia to prediabetes to T2D [17]. In parallel, a recent 6 year follow-up study showed that the severity of CAN is predictive of major adverse cardiovascular events [118]. In particular, an increase in cardiac autonomic composite score is accompanied by an elevated risk of cardiovascular death, nonfatal myocardial infarction, and nonfatal stroke. This provides evidence that meta-inflammation promotes cardiovascular complications possibly through worsening cardiac autonomic control. Hence, it becomes necessary to develop therapeutic interventions targeted at reducing inflammatory load in an attempt to reduce the burden of CAN and its associated cardiovascular risk.

## 8. Therapeutic Interventions to Ameliorate CAN in T2D

Studies investigating the ameliorative effect of antidiabetic treatment, with drugs such as metformin and pioglitazone, on CAN revealed that it is mediated by their anti-inflammatory properties [99,119,120]. In fact, metformin was demonstrated to improve cardiac autonomic function in nondiabetic-related inflammatory states such as hypertension [121]. Metformin treatment alleviated not only vascular inflammation by reducing the tissue expression of cyclooxygenase 2 (COX2) and NADPH oxidase 2 (NOX2), but also systemic inflammation demonstrated by a drop in TNF-α levels [121]. Treatment with metformin or pioglitazone was shown consistently to reduce adipose tissue inflammation and oxidative stress not only in cardiovascular tissues, but also in several brain regions including the brainstem at different stages of metabolic disease including prediabetes and T2D [84,99,100,102]. In these studies, either drug produced the desirable effects in nonhypoglycemic doses. In fact, the modulatory effect on adipose inflammation and CAN cannot be attributed to a reduction in blood glucose levels even in T2D. Indeed, tight glycemic control of T2D rats did not reduce markers of systemic inflammation and failed to reverse signs of CAN [84], potentially ascribing the beneficial effects of metformin and pioglitazone to pleiotropic effects. Specifically, their anti-inflammatory properties mediated by the activation of 5′ adenosine monophosphate-activated protein kinase and peroxisome proliferator-activated receptor gamma, respectively, were shown to ameliorate adipose tissue inflammation triggered by increased caloric intake [122,123,124]. Additionally, 5′ adenosine monophosphate-activated protein kinase activation has been implicated in desirable effects on autophagy with specific positive effects on diabetic neuropathy [84,125].

Along the same lines, drugs with a presumably neutral effect on blood glucose but proven to curb inflammation, such as minocycline, were shown to bring about an improvement in diabetic CAN [126]. Interestingly, minocycline has been shown to be an inhibitor of microglial activation, providing further evidence that central neuroinflammation might be involved in the pathogenesis of CAN in metabolic disorders. Significantly, minocycline was shown to normalize the alteration in GM and the ensuing proinflammatory gene expression resulting from high-fat diet feeding in rats [127] further emphasizing the interplay among these pathways in the development of CAN in the context of metabolic dysfunction. Similarly, cardiac autonomic manifestations in T2D normotensive patients were shown to favorably respond to ACE inhibition [128,129,130]. Treatment with an ACE inhibitor was shown to curb systemic inflammation and oxidative stress, as a consequence of normalized sympathetic overactivity, demonstrated by myocardial adrenergic innervation in this patient population [128]. Interestingly, not only could this observation be attributed to improved vascular elasticity [131] due to a reduced vascular inflammatory and oxidative milieu and, thus, enhanced BRS, but it could also be explained in light of an effect on adipose tissue inflammation. Indeed, ACE activity and angiotensin-II production are upregulated in metabolic disorders involving adipose tissue expansion and dysfunction [132,133]. Furthermore, the beneficial effect of ACE inhibitors could be related to reducing central inflammatory changes driven by systemic inflammation [12,84]. Moreover, the positive effect seen with Ang II inhibition could possibly be due to the reduction in hs-CRP associated with such a treatment [134], especially in the context of T2D where the levels of hs-CRP demonstrated a correlation with cardiac autonomic function [135].

Lastly, it might seem prudent to develop interventions tailored to reverse adipose tissue inflammation as a possible therapy for CAN. Indeed, such interventions were proposed for various cardiac and vascular disorders associated with metabolic impairment [33]. While the search for such tools is ongoing, simple interventions leading to this outcome might include modification of caloric intake. Indeed, previous research showed that approaches involving caloric restriction and intermittent fasting were associated with an ameliorative effect on both perivascular adipose inflammation and CAN [99,136].

## 9. Conclusions

This review attempted to provide a mechanistic and temporal framework, whereby the progression of low-grade inflammation from being localized in adipose tissue to a more systemic and central fashion parallels and potentially induces CAN deterioration during the natural course of the metabolic insult leading to T2D. Indeed, evidence supports the involvement of pro-oxidative and proinflammatory processes in the pathogenesis of CAN in T2D being adipose, vascular, systemic, and neuronal in nature. These have been shown to be related to hormonal disturbances such as hyperinsulinemia and an overactivation of the RAAS secondary to disease components. Additionally, metabolically induced alterations in GM were suggested to possibly mediate the effect of inflammation on CAN initiation and evolution. A detailed structured examination of these pathways in the future will assist in the development of targeted disease-modifying interventions to halt or reverse the manifestations of CAN in this patient population.

## Figures and Tables

**Table 1:** The Valsalva maneuver: expected responses in healthy individuals [8].

Phase	Maneuver	Hemodynamic Change
1	Onset of forced expiration	BP increases HR decreases (reflex)
2—shortest R–R interval	Continued forced exhalation	BP increases Reflex tachycardia (R–R decreases), SNS activation
3	Release of forced expiration	BP decreases HR increases
4—longest R–R interval	Continued release of forced expiration	BP increases Reflex bradycardia (R–R increases), PSNS activation

BP: blood pressure, HR: heart rate, PSNS: parasympathetic nervous system, SNS: sympathetic nervous system.

**Table 2:** Heart rate variability parameters. BRS, baroreceptor/reflex sensitivity.

Heart Rate Variability (HRV)	Description	Clinical Significance
Time-domain analysis [2] standard deviations (SDs)	**SD:** SD of N–N intervals	Overall
	**SDNN:** SD of *difference* between successive N–N intervals	Overall autonomic function
	**SDANN:** SD of *average* of differences between successive N–N intervals	Overall autonomic function
	**percentNN50:** percentage occurrence of N–N intervals above 50 ms	Parasympathetic function
	**RMSSD:** *Root mean square* of the differences between successive N–N intervals	Parasympathetic function
Valsalva ratio	$\frac{\mathrm{longest}\;R-R\;\mathrm{interval}}{\mathrm{shortest}\;R-R\;\mathrm{interval}}$	*Longest*: after release of the maneuver (phase 4: reflex bradycardia, parasympathetic) *Shortest*: during the maneuver (phase 2: reflex tachycardia, sympathetic)
Frequency-domain analysis	Very low frequency (VLF)	Sympathetic
	Low frequency (LF)	Parasympathetic and sympathetic, reflective of BRS
	High frequency (HF)	Parasympathetic
	Low/high frequency ratio (LF/HF)	Sympatho-vagal balance

**Table 3:** Measures of insulin *secretion* and *sensitivity.*

Parameter	Test	Methodology
Insulin secretion [2] (hypo- vs. hyperinsulinemia)	ΔC peptide	Before and 6 min after glucagon intravenous (IV) injection
	Fasting C-peptide	
	Stimulated C-peptide	
Insulin sensitivity: hyperinsulinemic–euglycemic clamp [2]	Incremental area under the curve (iAUC)	
Whole-body insulin sensitivity	M-value [2]	
Whole-body insulin resistance	Homeostatic model assessment for insulin resistance (HOMA-IR) [11,74]	At basal state
Oral glucose tolerance test [18]	OGTT	2 h after oral administration of 75 g of glucose
Insulin sensitivity: frequently sampled intravenous glucose tolerance test [18]	FSIVGTT	

**Table 4:** Glycemic status [8,91].

**Normal glucose tolerance (NGT)**	FPG < 5.6 mmol/L (100 mg/dL) 2 h PG < 7.8 mmol/L (<140 mg/dL)
**Isolated impaired fasting glucose (iIFG)**	FPG: 5.6–6.9 mmol/L (100–125 mg/dL) 2 h PG < 7.8 mmol/L (<140 mg/dL)
**Impaired glucose tolerance (IGT)**	FPG < 7.0 mmol/L (126 mg/dL) 2 h PG: 7.8–11.1 mmol/L (140–199 mg/dL)
**Newly diagnosed diabetes (NDD)**	FPG ≥ 7.0 mmol/L (126 mg/dL) 2 h PG ≥ 11.1 mmol/L (≥200 mg/dL)
